# Assessment of the interlaboratory variability and robustness of *JAK2*^V617F^ mutation assays: A study involving a consortium of 19 Italian laboratories

**DOI:** 10.18632/oncotarget.15940

**Published:** 2017-03-06

**Authors:** Margherita Perricone, Francesca Palandri, Emanuela Ottaviani, Mario Angelini, Laura Bagli, Enrica Bellesia, Meris Donati, Donato Gemmati, Patrizia Zucchini, Stefania Mancini, Valentina Marchica, Serena Trubini, Giovanna De Matteis, Silvia Di Zacomo, Mosè Favarato, Annamaria Fioroni, Caterina Bolzonella, Giorgia Maccari, Filippo Navaglia, Daniela Gatti, Luisa Toffolatti, Linda Orlandi, Vèronique Laloux, Marco Manfrini, Piero Galieni, Barbara Giannini, Alessia Tieghi, Sara Barulli, Maria Luisa Serino, Monica Maccaferri, Anna Rita Scortechini, Nicola Giuliani, Daniele Vallisa, Massimiliano Bonifacio, Patrizia Accorsi, Cristina Salbe, Vinicio Fazio, Milena Gusella, Eleonora Toffoletti, Marzia Salvucci, Mirija Svaldi, Filippo Gherlinzoni, Francesca Cassavia, Francesco Orsini, Giovanni Martinelli

**Affiliations:** ^1^ Department of Experimental, Diagnostic and Specialty Medicine, Institute of Hematology ‘L. and A. Seràgnoli’, University of Bologna, S. Orsola-Malpighi Hospital, Bologna, Italy; ^2^ Molecular Hematology Laboratory U.O.C of Hematology Hospital Mazzoni, Ascoli Piceno, Italy; ^3^ Medical Genetics Unit- Hub Laboratory AUSL Romagna, Pievesestina di Cesena, Italy; ^4^ Imaging and Laboratory Diagnostic Department, Clinical Chemistry and Endocrinology Laboratory, Hematology Unit, Oncology and Technology Department, Hospital S. Maria Nuova, IRCCS, Reggio Emilia, Italy; ^5^ Clinical Pathology Laboratory, A.O. Ospedali Riuniti Marche Nord, Pesaro, Italy; ^6^ Center Hemostasis and Thrombosis, Section of Medical Biochemistry, Molecular Biology and Genetics, Department of Biomedical and Specialty Surgical Sciences, University of Ferrara, Ferrara, Italy; ^7^ Department of Medical and Surgical Sciences, Division of Hematology, University of Modena and Reggio Emilia, Modena, Italy; ^8^ Clinical Hematology Laboratory, Department of Molecular and Clinical Sciences, Polytechnic University of Marche, Ancona, Italy; ^9^ Department of Clinical and Experimental Medicine, University of Parma, Parma, Italy; ^10^ Clinical Pathology, Molecular Biology Laboratory, and Hematology/Bone Marrow Transplantation Unit, AUSL Piacenza, Piacenza, Italy; ^11^ Section of Clinical Biochemistry and Section of Hematology, Azienda Ospedaliera Universitaria Integrata di Verona, Verona, Italy; ^12^ Department of Hematology, Blood Bank and Biotechnology, Ospedale Civile Pescara, Pescara, Italy; ^13^ UOS Molecular Diagnostics, Department of Clinical Pathology, ULSS12 Venetian, Venice, Italy; ^14^ UOC laboratory medicine, P.O. San Salvatore, Sulmona, L’Aquila, Italy; ^15^ Department of Oncology, Laboratory of Pharmacology and Molecular Biology, ULSS 18, Rovigo, Italy; ^16^ Clinical Hematology, Department of Experimental and Clinical Medical Sciences, University of Udine, Udine, Italy; ^17^ Department of Laboratory Medicine, University-Hospital of Padova, Padova, Italy; ^18^ Department of Haematology and BMT, Healthcare Company of South Tyrol, District of Bolzano, Bolzano, Italy; ^19^ Department of Pathology and Haematology, Treviso General Hospital, Treviso, Italy; ^20^ Werfen, Milano, Italy; ^21^ QIAGEN GmbH, Hilden, Germany, member of the European LeukemiaNet (ELN) Foundation Circle

**Keywords:** JAK2 V617F mutation, myeloproliferative neoplasms, qPCR standardization, molecular diagnosis

## Abstract

To date, a plenty of techniques for the detection of *JAK2*^V617F^ is used over different laboratories, with substantial differences in specificity and sensitivity. Therefore, to provide reliable and comparable results, the standardization of molecular techniques is mandatory.

A network of 19 centers was established to 1) evaluate the inter- and intra-laboratory variability in *JAK2*^V617F^ quantification, 2) identify the most robust assay for the standardization of the molecular test and 3) allow consistent interpretation of individual patient analysis results. The study was conceived in 3 different rounds, in which all centers had to blindly test DNA samples with different *JAK2*^V617F^ allele burden (AB) using both quantitative and qualitative assays.

The positivity of samples with an AB < 1% was not detected by qualitative assays. Conversely, laboratories performing the quantitative approach were able to determine the expected *JAK2*^V617F^ AB. Quantitative results were reliable across all mutation loads with moderate variability at low AB (0.1 and 1%; CV = 0.46 and 0.77, respectively). Remarkably, all laboratories clearly distinguished between the 0.1 and 1% mutated samples.

In conclusion, a qualitative approach is not sensitive enough to detect the *JAK2*^V617F^ mutation, especially at low AB. On the contrary, the ipsogen *JAK2* MutaQuant CE-IVD kit resulted in a high, efficient and sensitive quantification detection of all mutation loads. This study sets the basis for the standardization of molecular techniques for *JAK2*^V617F^ determination, which will require the employment of approved operating procedures and the use of certificated standards, such as the recent WHO 1st International Reference Panel for Genomic *JAK2*^V617F^.

## INTRODUCTION

The *JAK2*^V617F^ mutation represents a hallmark of Philadelphia (Ph)- negative myeloproliferative neoplasms (MPNs), fulfilling a 2008 World Health Organization (WHO) major criterion for the diagnosis of MPNs [1–2]. The *JAK2*^V617F^ mutation is an acquired, somatic mutation carried by almost all patients (approximately 95%) with polycythemia vera (PV) and in more than half (approximately 50–60%) of those with essential thrombocythemia (ET) or primary myelofibrosis (PMF) [3, 4].

The assessment of the *JAK2*^V617F^ allele burden (AB) is a common practice either at diagnosis, for prognostic information, or during treatment as a means to assess minimal residual disease [5]. Indeed, *JAK2*^V617F^ AB seems to be correlated with an increased risk of thrombosis and evolution in a secondary myelofibrosis in PV (PPV-MF) and, possibly, in ET (PET-MF) [6, 7]. Additionally, low AB is associated with a reduced survival in PMF [8–11]. With regard to drug therapy, several studies showed that interferon-alpha, and the most recent telomerase inhibitors (Imetelstat), significantly reduces *JAK2*^V617F^ mutation burden, whereas, *JAK* inhibitors and hydroxyhurea (HU) did not have any significant effects [12–21]. Moreover, *JAK2*^V617F^ quantification has been incorporated as a potentially useful tool to predict relapse in those patients who underwent allogeneic stem-cell transplantation (alloHSCT). In this setting of patients, early monitoring of the AB (1, 3 and 6 months post alloHSCT) is crucial to predict overall survival and risk of relapse and might guide therapeutic decisions [22–24].

To date, a plenty of techniques for *JAK2*^V617F^ determination is used over different laboratories, with substantial differences in specificity and sensitivity [5, 25–31]. The extensive and worldwide use of molecular techniques with high sensitivity has significantly increased our ability to detect small mutated clones, with low AB (i.e. <1% of mutation loads) [5, 26, 32]. Additionally, many recent studies have shown that a small clonal hematopoiesis may be present also in otherwise healthy subjects at low level (0.03–1%) [27, 32–38]. In the context of highly sensitive allele-specific assays and low mutant AB in the peripheral blood, the possibility of both false-positive and false-negative test results is not negligible [5, 32].

Therefore, to provide a reliable and comparable molecular results, the standardization of molecular techniques is urgently needed. In a recent study by European LeukemiaNet/MPN&MPNr-EuroNet group, nine different *JAK2*^V617F^ quantitative assays were evaluated by the 12 participant laboratories, with the aim to identify the most robust one for routine diagnostic purpose and also for post alloHSCT monitoring [39]. Therefore, a network of 19 Italian laboratories was established with the aim 1) to evaluate the inter- and intra-laboratory variability in *JAK2*^V617F^ quantification in these 19 centers, 2) to identify the most robust assay for the standardization of the molecular test and 3) to allow consistent interpretation of individual patient analysis results.

## RESULTS

**Figure 1:**
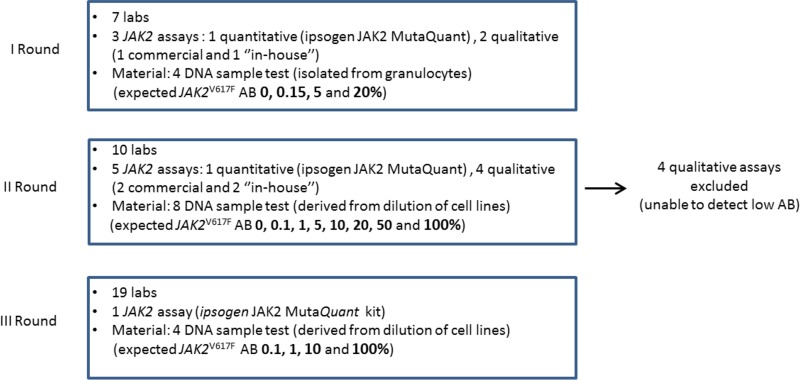
Design of the study A network of 19 Italian Centers was established and the study was conceived in 3 different rounds, in which seven, ten and nineteen laboratories participated, respectively. Each laboratory had to blindly test DNA samples with different *JAK2*^V617F^ allele burden (AB). Overall, one quantitative and four qualitative assays were evaluated.

Between 2014 and 2015, a network of 19 Italian laboratories, routinely involved in the molecular diagnosis of MPNs, was established. The study was coordinated by the Institute of Hematology “L. e A. Seràgnoli”, Bologna, and conceived in 3 different rounds in which seven, ten and nineteen laboratories were included over time, respectively (Figure 1). Overall, one quantitative (ipsogen *JAK2* MutaQuant kit, QIAGEN) and four qualitative assays were evaluated. Of these latter, two were commercial (ipsogen *JAK2* MutaSearch kit, QIAGEN, and GeneQuality JAK-2, AB Analitica) and two were built “in-house” methods: allele specific polymerase chain reaction (AS-PCR) and Amplification-refractory mutation system (ARMS) analysis [25, 30].

### I Round: proficiency test

In order to obtain information about the variability in *JAK2*^V617F^ quantification between different centers, seven laboratories were employed to evaluate several DNA samples with their own established *JAK2*^V617F^ qualitative and/or quantitative method. Precisely, four DNA samples derived from granulocytes of patients with a diagnosis of MPNs were analyzed. All laboratories using the quantitative assay (ipsogen *JAK2* MutaQuant kit) were able to determine the expected *JAK2*^V617F^ AB, as summarized in Table 1. Only in one case (i.e. DNA sample 1), the Center 3 obtained a false positive result. Indeed, the sample was found to be positive with an AB of 0.13%. On the contrary, two Centers (i.e. 6 and 7), using a qualitative approach, were not able to detect the positivity of the DNA sample 2 (with an expected AB of 0.15%).

**Table 1:** I Round *JAK2*^V617F^ mutation burden and summary statistics (minimum, maximum, calculation of median, coefficient of variation) of results obtained by the seven participating laboratories, using their everyday analysis method

Sample	Expected JAK2V617F AB (%)	C1	C2	C3	C4	C5	Min	Median	Max	CV (Coefficient of Variation)	C6	C7
DNA 1	0.005	0.0056	0.00013	0.13	0	0.0007	0	0.00315	0.13	2.1	Neg	Neg
DNA 2	0.15	0.18	0.13	0.19	0.15	0.15	0.13	0.15	0.19	0.15	Neg	Neg
DNA 3	5	7.84	4.19	6.2	8.22	6.25	4.19	6.225	8.22	0.24	Pos	Pos
DNA 4	20	23.17	25.95	20.24	19.44	19.82	19.44	20.03	25.95	0.08	Pos	Pos

The expected *JAK2*^V617F^ allele burden (AB, %) was 0.005 (DNA 1), 0.15 (DNA 2), 5 (DNA 3) and 20 (DNA 4). Centers 1–5 performed quantitative assay (ipsogen JAK2 MutaQuant kit) whereas Centers 6 and 7 used a qualitative approach: allele specific polymerase chain reaction (AS-PCR) and ipsogen JAK2 MutaSearch kit, respectively.

### II Round: comparison between molecular assays

With the aim to further investigate on the inter-laboratory variability in quantifying *JAK2*^V617F^ mutation, especially at low mutation burden, a second standardization round was developed and three additional laboratories were included. Eight DNA samples, derived from dilution of cell lines negative and positive for the *JAK2*^V617F^ mutation, were tested by each laboratory with both ipsogen *JAK2* MutaQuant kit and their own routine qualitative or quantitative method.

We first examined the methods sensitivity, and the detection ability of the ipsogen *JAK2* MutaQuant kit at low-positive samples (i.e. 0.1 and 1%) was compared to those of qualitative *JAK2* commercial and validated “in-house” methods. Overall, the ARMS-PCR “in-house” method and the ipsogen *JAK2* MutaSearch kit were able to detect the positivity of the sample with AB of 1%, whereas none of the laboratories using any qualitative methods were able to detect the low-positive sample (i.e. AB < 0.1%). Remarkably, laboratories using the quantitative approach clearly defined the positivity of both 1% and 0.1% mutated samples. Specifically, 10 out of 16 *JAK2*^V617F^ determinations were clearly defined positive, with an AB ≥ 0.091% which is the Limit Of Detection (LOD) of the ipsogen *JAK2* MutaQuant kit. In the remaining 6 cases, the *JAK2*^V617F^ mutation percentage was found between Limit of Blank (LOB = 0.014%) and LOD.

**Figure 2:**
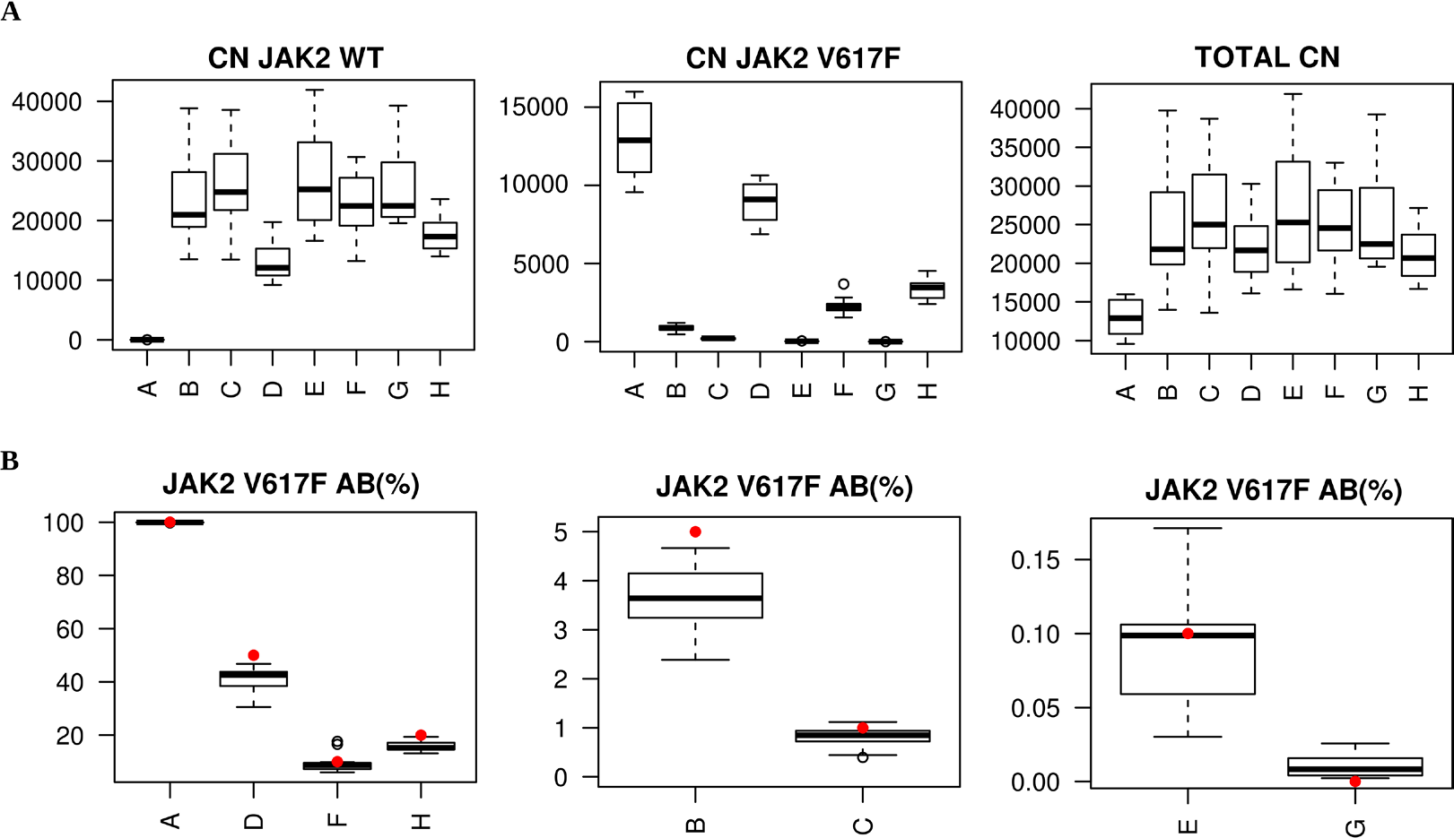
II Round ipsogen *JAK2* MutaQuant kit Copy number (CN) boxplot of *JAK2* wild-type (WT), *JAK2* mutated (V617F) and *JAK2* total (**A**) and *JAK2*^V617F^ mutation percentage boxplot (**B**) for each of the 8 DNA samples. Red dots are expected values.

Additionally, the inter-laboratory variability evaluation was restricted to the ipsogen *JAK2* MutaQuant kit, as six out of 10 participating laboratories already used this assay in their routine practice. The data from two laboratories (i.e. Centers 3 and 8) were excluded from statistical analysis, as Negative Controls of *JAK2*^V617F^ mutation (NC-VF) were found to be positive (> 0.1%) for each run, and considered as invalid runs. This was mainly due to either operator error or to instrumentation suitability, instead of an intrinsic bias of the kit. Overall, quantitative results between the laboratories were reliable as summarized in Table 2 and showed in Figure 2. A small variability was observed especially at low AB (0.1 and 1%, CV = 0.42 and 0.24, respectively).

**Table 2:** II Round *JAK2*^V617F^ mutation burden and summary statistics (minimum, maximum, calculation of median, coefficient of variation) of results obtained by the ten participating laboratories with ipsogen *JAK2* MutaQuant kit

Sample	Expected *JAK2*^V617F^AB (%)	Min.	Median	Max	CV (Coefficient of Variation)
**DNA G**	0	0.002	0.008234	0.02	0.73
**DNA E**	0.1	0.03	0.09874	0.17	0.42
**DNA C**	1	0.39	0.8471	1.12	0.24
**DNA B**	5	2.38	3.643	4.66	0.18
**DNA F**	10	6.04	8.794	17.65	0.38
**DNA H**	20	13.12	15.2	19.35	0.11
**DNA D**	50	30.54	42.62	46.76	0.12
**DNA A**	100	99.76	99.92	99.97	0.0005

The expected *JAK2*^V617F^ mutation burden of the eight DNA samples was 0, 0.1, 1, 10, 20, 50 and 100%.

### III Round: inter-laboratory standardization by ipsogen JAK2 MutaQuant kit

The study was extended to 9 additional centers to confirm the robustness of the ipsogen *JAK2* MutaQuant kit in a larger cohort. Each participating laboratory had to test four DNA samples, derived from dilution of cell lines as described above, only with ipsogen *JAK2* MutaQuant kit.

**Figure 3:**
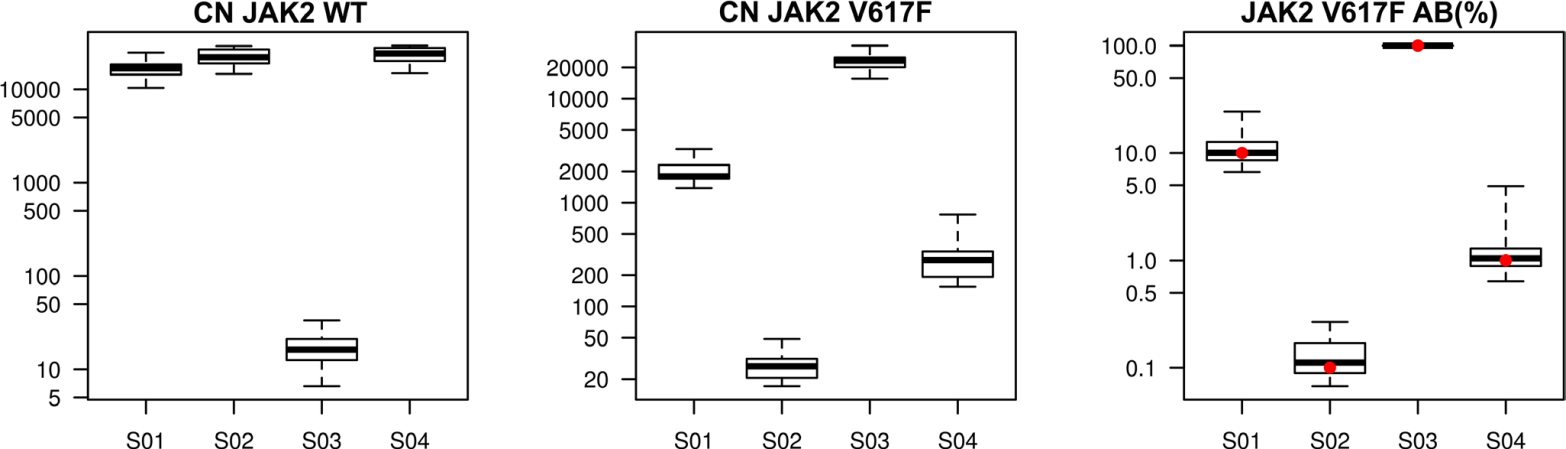
III Round ipsogen *JAK2* MutaQuant kit Copy number (CN) boxplot of *JAK2* wild-type (WT), *JAK2* mutated (V617F) and *JAK2*^V617F^ mutation percentage boxplot for each of the 4 DNA samples. Red dots are expected values.

The quantification data from Center 11 were excluded from statistical analysis, as NC-VF was found to be positive (> 0.1%) for each run and Positive Control of *JAK2*^V617F^ mutation (PC-VF) did not reach the recommended value (> 99.9%). Moreover Center 9 failed to perform correctly both runs due to instrument failure. Of note, both Center 9 and Center 11 did not assess *JAK2*^V617F^ AB in their own routine practice. Among the remaining laboratories, Centers 2, 6 and 17 did not reach the minimum number of *JAK2* total copy number required (10.000 copies) in five different determinations (two at 0.1% AB sample, two at 1% and one at 10%, respectively), and, therefore, these points were not included in the analysis. Quantitative results were reliable across all mutation loads, as reported in Table 3 and showed in Figure 3. All the 17 laboratories were able to quantify the 0.1% AB sample with the same variability observed in the previous II round (CV = 0.46 and 0.42, respectively). Specifically, 23 out of 32 *JAK2*^V617F^ determinations were clearly defined positive, with an AB > 0.091% (LOD). In the remaining 9 cases, the *JAK2*^V617F^ mutation percentage was found between LOB and LOD. Surprisingly, a higher variability between laboratories was observed at 1% of AB (CV = 0.77, *vs* 0.24 in the II round). More importantly, all laboratories clearly distinguished between the 0.1 and 1% mutated samples.

**Table 3:** III Round *JAK2*^V617F^ mutation burden and summary statistics (minimum, maximum, calculation of median, coefficient of variation) of results obtained by the nineteen participating laboratories with ipsogen *JAK2* MutaQuant kit

Sample	Expected *JAK2*^V617F^AB (%)	Min.	Median	Max	CV (Coefficient of Variation)
**S02**	0.1	0.07	0.11	0.27	0.46
**S04**	1	0.64	1.05	4.90	0.77
**S01**	10	6.67	10.04	24.37	0.37
**S03**	100	99.79	99.92	99.97	0.0005

The expected *JAK2*^V617F^ mutation burden of the four DNA samples was 0.1, 1, 10 and 100%.

We also evaluated the robustness of the quantitative approach in terms of amplification efficiency. It is well known that amplification efficiency in PCR measures the amount of template converted into amplified product during each cycle of the exponential phase of the reaction. At 100% efficiency, the quantity of product exactly doubles each cycle, thus an efficiency close to 100% is the best indicator of a robust, reproducible assay. An amplification efficiency of 90–105% is recommended for each assay. In our study, the mean value of amplification efficiency obtained, with respect to all runs performed, was of 92% with a CV value of 0.065, confirming the sensitivity and the robustness of the ipsogen *JAK2* MutaQuant kit.

## DISCUSSION AND CONCLUSIONS

In this study we demonstrated that a qualitative approach is not sensitive enough to detect *JAK2*^V617F^ mutation at low mutation burden (i.e. < 1%). Conversely, the quantitative approach proved to be highly efficient and sensitive, although a modest variability was observed between all participating centers, both in the II and in the III round (CV = 0.42 and 0.46, respectively). Interestingly, only the qualitative ARMS-PCR methods (both “in-house” and ipsogen *JAK2* MutaSearch kit) and the quantitative approach were able to detect the positivity of samples with and AB of 1%. An acceptable variability was observed at this AB in the II round (CV = 0.24) whereas a higher inter-laboratory variability was registered in the III one (CV = 0.77). With regard to samples with AB > 1%, a lower inter-laboratory variability was observed, as demonstrated by CV which ranges from a minimum of 0.0005 to a maximum of 0.38.

Overall, the quantitative ipsogen *JAK2* MutaQuant kit assay performed consistently across different platforms, affirming itself as a robust method to obtain comparable results. This is confirmed by optimal amplification efficiency obtained from each laboratory involved in the study. Indeed, the mean efficiency obtained in this study was of 92% with a CV of 0.065. The observed variability can be explained with both differences in laboratory experience in *JAK2*^V617F^ quantitative determination and intrinsic instrumental bias, as happened in our study, with some laboratories experiencing technical issues. This, together with the variability observed at low AB samples, highlights the need for the standardization of practices, including both pre-analytical and analytical phases. To this aim, the WHO 1st International Reference Panel for Genomic JAK2 V617F (WHO document WHO/BS/2016.2293) was established in 2016 by the Expert Committee on Biological Standardization of the World Health Organization [40]. The availability of *JAK2*^V617F^ primary standards should improve the quality of MPN genomic diagnostics by enabling the calibration of assays and kits, and the derivation of secondary standards for routine diagnostic use in determining testing accuracy and sensitivity, thus providing inter-laboratory comparison towards the harmonization of *JAK2*^V617F^ testing.

Moreover, we are considering evaluating digital PCR (dPCR). This emerging technology may improve the ability to detect rare mutations and/or low-positive samples due to higher sensitivity and precision, especially during follow-up to assess minimal residual disease or to monitor patients post alloHSCT [41–42]. But further studies are needed on this technology to reach the level of standardization of real-time qPCR.

In conclusion, this study sets the basis for the standardization of molecular techniques for *JAK2*^V617F^ determination which will require the employment of approved operating procedures and the use of certificated standards to calibrate *JAK2*^V617F^ quantitative assays.

## MATERIALS AND METHODS

The study was conceived in 3 different rounds, in which 19 Italian laboratories were employed. Centers 9 and 16 did not perform *JAK2*^V617F^ molecular testing in their routine. Of the remaining centers, 8 used only a quantitative approach (2 “in-house” and 1 commercial assays), 7 performed only a qualitative evaluation (1 “in-house” and 3 commercial assay) of *JAK2*^V617F^ mutation whereas 2 laboratories used both qualitative and quantitative approaches (2 “in-house” and 1 commercial assays). Regarding real-time PCR instruments used during the study: most (13 out of 19) of the laboratories used Applied Biosystem platforms (ABI7300/7500/7900; Applied Biosystem, Foster City, CA, USA); two laboratories used a Lightcycler LC480 platform (Roche Applied Science, Penzberg, Germany), and the remaining four laboratories used a Rotor-Gene Q 2plex/MDx 5plex HRM instrument (QIAGEN GmbH, Hilden, Germany) (Table 4).

**Table 4:** Summary of participating centers, methods used in their routine practice for *JAK2*^V617F^ detection and instrumentation used in their routine and/or in the study (specified when different)

ID Center		Method	Instrumentation
**1**	Quantitative	ipsogen JAK2 MutaQuant kit	Applied Biosystem 7900HT Fast* Real-Time PCR System
**2**	Quantitative	ipsogen JAK2 MutaQuant kit	Applied Biosystems 7300 Real-Time PCR System
**3**	Quantitative	ipsogen JAK2 MutaQuant kit	Rotor-Gene Q 2plex HRM System Rotor-Gene Q MDx 5plex HRM System (3rd Round)
**4**	Quantitative	ipsogen JAK2 MutaQuant kit	Applied Biosystems 7300 Real-Time PCR System
**5**	Qualitative home-made Quantitative	ARMS PCR (Chen et al., 2007) [30] ipsogen JAK2 MutaQuant kit	7500 Real Time PCR System Life Technologies
**6**	Qualitative home-made	Allele-specific PCR (Baxter et al, Lancet 2005)[25]	LightCycler 480 (2nd and 3rd Round)
**7**	Qualitative	ipsogen JAK2 MutaSearch	Applied Biosystems 7300 Real-Time PCR System
**8**	Qualitative	AB Analitica GeneQuality JAK-2 kit	Applied Biosystems 7300 Real-Time PCR System
**9**	N.A.	N.A.	LightCycler 480 (2nd and 3rd Round)
**10**	Quantitative	ipsogen JAK2 MutaQuant kit	Applied Biosystems 7500 Fast* Real-time PCR System
**11**	Qualitative home-made	Allele-specific PCR (Baxter et al, Lancet 2005)[25]	Rotor-Gene Q MDx 5plex HRM (3rd Round)
**12**	Quantitative	ipsogen JAK2 MutaQuant kit	Rotor-Gene Q MDx 5plex HRM
**13**	Quantitative home-made	Allele-specific primers (0,1-25%) Allele-specific hydrolysis probes (10–90%)	ABI PRISM 7900 HT Real-Time PCR System
**14**	Qualitative home-made Quantitative	Allele-specific PCR (Baxter et al, Lancet 2005)[25] ipsogen JAK2 MutaQuant kit	Applied Biosystems 7500 Real-time PCR System
**15**	Qualitative	ipsogen JAK2 MutaScreen	Applied Biosystems 7500 Fast* Dx PCR System
**16**	N.A.	N.A.	Applied Biosystems 7500 Fast* Real-time PCR System
**17**	Quantitative home-made	Allele-specific PCR (Søren Germer et al, Genome Res. 2000)	ABI PRISM 7900 HT SDS
**18**	Qualitative	ipsogen JAK2 MutaScreen	Applied Biosystems 7500 Real-time PCR System
**19**	Qualitative	ipsogen JAK2 MutaScreen	StepOnePlus Real-Time PCR System Rotor-Gene Q MDx 5plex HRM (3rd Round)

* Fast mode was not used, as the ipsogen *JAK2* MutaQuant kit is not compatible with “Fast” mode.

### I Round

In the I round, we aimed to investigate the inter-laboratory variability on different mutation loads. In this first step, seven laboratories were involved (Center 1–7). Four of them routinely performed quantitative analysis of *JAK2*^V617F^ with ipsogen *JAK2* MutaQuant Kit (QIAGEN), two used a qualitative approach for *JAK2*^V617F^ evaluation (1 “in-house” method, 1 ipsogen *JAK2* MutaScreen Kit - QIAGEN) whereas one laboratory (Center 5) assessed both qualitative and quantitative assays. Each center had to test four DNA samples (DNA 1–4) with the method routinely employed in their own laboratory. DNA samples were isolated from granulocytes of patients with diagnosis of MPNs. The expected mutation burden of the 4 DNA samples was 0.005 (DNA 1), 0.15 (DNA 2), 5 (DNA 3) and 20% (DNA 4), as previously quantified by Bologna's laboratory with ipsogen *JAK2* MutaQuant Kit.

All patients provided an informed written consent in accordance with the Declaration of Helsinki for the use of remnant DNA for investigational purposes. The study was approved by the local Ethics Committee.

### II Round

To further investigate the inter-laboratory variability on low-positive samples, a II round was developed. The two main objectives of this round were to assess inter-laboratory variability across the 10 participating clinical centers and to compare the low-positive sample detection ability of the ipsogen *JAK2* MutaQuant kit with the *JAK2* validated “in-house” methods. Compared to the first round, three additional centers (Centers 8–10) were included in this step: only Center 8 and 10 routinely performed *JAK2*^V617F^ evaluation (1 with a qualitative and 1 with a quantitative method, respectively). Eight DNA test samples (DNA Samples A-H) were manufactured by QIAGEN and were centrally distributed by Werfen. The DNA samples were derived from dilution of cell lines: K562 (*JAK2*^V617F^ negative) and MUTZ-8 (*JAK2*^V617F^ positive). The ipsogen *JAK2* MutaQuant kits and associated master-mix were provided by QIAGEN and Werfen. Each center had to blindly test the DNA samples in four different runs: 2 runs were performed with the ipsogen *JAK2* MutaQuant kit and 2 runs with their validated qualitative or quantitative method. The expected mutation burden of the DNA samples was 0, 0.1, 1, 10, 20, 50 and 100%.

### III Round

The III round was intended to confirm the robustness of the ipsogen *JAK2* MutaQuant kit. Qualitative methods were therefore excluded and the study was extended to 9 additional laboratories (Centers 11–19). Centers 12, 13 and 17 routinely assessed quantitative evaluation of JAK2^V617F^ mutation (2 “in-house” and 1 commercial methods), 4 laboratories (Centers 11, 15, 18 and 19) used qualitative assays (1 “in-house” and 1 commercial methods), Center 14 performed both qualitative (“in-house”) and quantitative (ipsogen *JAK2* MutaQuant kit) analysis whereas Center 16 did not perform *JAK2*^V617F^ molecular testing in its routine. Four DNA test samples (DNA Samples S01-S04), provided from the same batches as II round's DNA Samples, were centrally distributed by Werfen. The ipsogen *JAK2* MutaQuant kits and associated master-mix were provided by QIAGEN and Werfen. Each laboratory had to blindly test the DNA samples in 2 runs with the ipsogen *JAK2* MutaQuant kit. The expected mutation burden of the four DNA samples was 0.1, 1, 10 and 100%.

Moreover, amplification efficiency (E) was calculated from the slope of the standard curve using the following formula: E = 10^−1/slope^. Amplification efficiency was expressed as a percentage, that is the percent of template that was amplified in each cycle. To convert E into a percentage we used the following formula: % Efficiency = (E – 1) × 100%.

### Data collection and run validity check

Raw data were collected and run validity was checked according to manufacturer's instructions in the kit's handbook.

### Statistical method

Statistical analysis was carried out by QIAGEN and by Bologna University. Wild type and mutation copy numbers together with mutation percentage were summarized by mean, median, first and third quartiles, standard deviation and coefficient of variation and plotted by sample for the ipsogen JAK2 MutaQuant kit.

### Inter-laboratory variability: II round

Fisher test was performed by sample to compare variance in order to conclude on the acceptability of the inter-laboratory variability.

### Inter-laboratory variability: III round

Shapiro-Wilk normality test was performed to check for data normality and quantile-quantile normal plots were employed for data visual inspection. Kruskall-Wallis test was applied and multiple comparison post-hoc test (Wilcoxon test) was carried out to identify the significant differences. False discovery rate correction was applied to avoid increase in type I error (false positive) because of multiple testing following the Benjamini and Hochberg procedure.

## References

[1] Tefferi A, Vardiman JW (2008). Classification and diagnosis of myeloproliferative neoplasms: the 2008 World Health Organization criteria and point-of-care diagnostic algorithms. Leukemia.

[2] Arber DA, Orazi A, Hasserjian R, Thiele J, Borowitz MJ, Le Beau MM, Bloomfield CD, Cazzola M, Vardiman JW (2016). The 2016 revision to the World Health Organization classification of myeloid neoplasms and acute leukemia. Blood.

[3] Nangalia J, Massie CE, Baxter EJ, Nice FL, Gundem G, Wedge DC, Avezov E, Li J, Kollmann K, Kent DG, Aziz A, Godfrey AL, Hinton J (2013). Somatic CALR mutations in myeloproliferative neoplasms with nonmutated JAK2. N Engl J Med.

[4] Klampfl T, Gisslinger H, Harutyunyan AS, Nivarthi H, Rumi E, Milosevic JD, Them NC, Berg T, Gisslinger B, Pietra D, Chen D, Vladimer GI, Bagienski K (2013). Somatic mutations of calreticulin in myeloproliferative neoplasms. N Engl J Med.

[5] Bench AJ, White HE, Foroni L, Godfrey AL, Gerrard G, Akiki S, Awan A, Carter I, Goday-Fernandez A, Langabeer SE, Clench T, Clark J, Evans PA (2013). British Committee for Standards in Haematology. Molecular diagnosis of the myeloproliferative neoplasms: UK guidelines for the detection of JAK2 V617F and other relevant mutations. Br J Haematol.

[6] Passamonti F, Rumi E, Pietra D, Elena C, Boveri E, Arcaini L, Roncoroni E, Astori C, Merli M, Boggi S, Pascutto C, Lazzarino M, Cazzola M (2010). A prospective study of 338 patients with polycythemia vera: the impact of JAK2 (V617F) allele burden and leukocytosis on fibrotic or leukemic disease transformation and vascular complications. Leukemia.

[7] Silver RT, Vandris K, Wang YL, Adriano F, Jones AV, Christos PJ, Cross NC (2011). JAK2(V617F) allele burden in polycythemia vera correlates with grade of myelofibrosis, but is not substantially affected by therapy. Leuk Res.

[8] Barosi G, Bergamaschi G, Marchetti M, Vannucchi AM, Guglielmelli P, Antonioli E, Massa M, Rosti V, Campanelli R, Villani L, Viarengo G, Gattoni E, Gerli G (2007). Gruppo Italiano Malattie Ematologiche Maligne dell’Adulto (GIMEMA) Italian Registry of Myelofibrosis. JAK2 V617F mutational status predicts progression to large splenomegaly and leukemic transformation in primary myelofibrosis. Blood.

[9] Tefferi A, Lasho TL, Huang J, Finke C, Mesa RA, Li CY, Wu W, Hanson CA, Pardanani A (2008). Low JAK2V617F allele burden in primary myelofibrosis, compared to either a higher allele burden or unmutated status, is associated with inferior overall and leukemia-free survival. Leukemia.

[10] Guglielmelli P, Barosi G, Specchia G, Rambaldi A, Lo Coco F, Antonioli E, Pieri L, Pancrazzi A, Ponziani V, Delaini F, Longo G, Ammatuna E, Liso V (2009). Identification of patients with poorer survival in primary myelofibrosis based on the burden of JAK2V617F mutated allele. Blood.

[11] Barosi G, Poletto V, Massa M, Campanelli R, Villani L, Bonetti E, Viarengo G, Catarsi P, Klersy C, Rosti V (2013). JAK2 V617F genotype is a strong determinant of blast transformation in primary myelofibrosis. PLoS One.

[12] Girodon F, Schaeffer C, Cleyrat C, Mounier M, Lafont I, Santos FD, Duval A, Maynadié M, Hermouet S (2008). Frequent reduction or absence of detection of the JAK2-mutated clone in JAK2V617F-positive patients within the first years of hydroxyurea therapy. Haematologica.

[13] Ricksten A, Palmqvist L, Johansson P, Andreasson B (2008). Rapid decline of JAK2V617F levels during hydroxyurea treatment in patients with polycythemia vera and essential thrombocythemia. Haematologica.

[14] Antonioli E, Carobbio A, Pieri L, Pancrazzi A, Guglielmelli P, Delaini F, Ponziani V, Bartalucci N, Tozzi L, Bosi A, Rambaldi A, Barbui T, Vannucchi AM (2010). Hydroxyurea does not appreciably reduce JAK2 V617F allele burden in patients with polycythemia vera or essential thrombocythemia. Haematologica.

[15] Besses C, Alvarez-Larrán A, Martínez-Avilés L, Mojal S, Longarón R, Salar A, Florensa L, Serrano S, Bellosillo B (2011). Modulation of JAK2 V617F allele burden dynamics by hydroxycarbamide in polycythaemia vera and essential thrombocythaemia patients. Br J Haematol.

[16] Kuriakose ET, Gjoni S, Wang YL, Baumann R, Jones AV, Cross NC, Silver RT (2013). JAK2V617F allele burden is reduced by busulfan therapy: a new observation using an old drug. Haematologica.

[17] Quintás-Cardama A, Abdel-Wahab O, Manshouri T, Kilpivaara O, Cortes J, Roupie AL, Zhang SJ, Harris D, Estrov Z, Kantarjian H, Levine RL, Verstovsek S (2013). Molecular analysis of patients with polycythemia vera or essential thrombocythemia receiving pegylated interferon α-2a. Blood.

[18] Stauffer Larsen T, Iversen KF, Hansen E, Mathiasen AB, Marcher C, Frederiksen M, Larsen H, Helleberg I, Riley CH, Bjerrum OW, Rønnov-Jessen D, Møller MB, de Stricker K (2013). Long term molecular responses in a cohort of Danish patients with essential thrombocythemia, polycythemia vera and myelofibrosis treated with recombinant interferon alpha. Leuk Res.

[19] Abdel-Wahab O, Pardanani A, Bernard OA, Finazzi G, Crispino JD, Gisslinger H, Kralovics R, Odenike O, Bhalla K, Gupta V, Barosi G, Gotlib J, Guglielmelli P (2012). Unraveling the genetic underpinnings of myeloproliferative neoplasms and understanding their effect on disease course and response to therapy: proceedings from the 6th International Post-ASH Symposium. Am J Hematol.

[20] Bjørn ME, de Stricker K, Kjær L, Ellemann K, Hasselbalch HC (2014). Combination therapy with interferon and JAK1–2 inhibitor is feasible: Proof of concept with rapid reduction in JAK2V617F-allele burden in polycythemia vera. Leuk Res Rep.

[21] Baerlocher GM, Oppliger Leibundgut E, Ottmann OG, Spitzer G, Odenike O, McDevitt MA, Röth A, Daskalakis M, Burington B, Stuart M, Snyder DS (2015). Telomerase Inhibitor Imetelstat in Patients with Essential Thrombocythemia. N Engl J Med.

[22] Alchalby H, Badbaran A, Zabelina T, Kobbe G, Hahn J, Wolff D, Bornhäuser M, Thiede C, Baurmann H, Bethge W, Hildebrandt Y, Bacher U, Fehse B (2010). Impact of JAK2V617F mutation status, allele burden, and clearance after allogeneic stem cell transplantation for myelofibrosis. Blood.

[23] Lange T, Edelmann A, Siebolts U, Krahl R, Nehring C, Jäkel N, Cross M, Maier J, Niederwieser D, Wickenhauser C (2013). JAK2 p.V617F allele burden in myeloproliferative neoplasms one month after allogeneic stem cell transplantation significantly predicts outcome and risk of relapse. Haematologica.

[24] Kröger N, Badbaran A, Holler E, Hahn J, Kobbe G, Bornhäuser M, Reiter A, Zabelina T, Zander AR, Fehse B (2007). Monitoring of the JAK2-V617F mutation by highly sensitive quantitative real-time PCR after allogeneic stem cell transplantation in patients with myelofibrosis. Blood.

[25] Baxter EJ, Scott LM, Campbell PJ, East C, Fourouclas N, Swanton S, Vassiliou GS, Bench AJ, Boyd EM, Curtin N, Scott MA, Erber WN, Green AR (2005). Cancer Genome Project. Acquired mutation of the tyrosine kinase JAK2 in human myeloproliferative disorders. Lancet.

[26] McClure R, Mai M, Lasho T (2006). Validation of two clinically useful assays for evaluation of JAK2 V617F mutation in chronic myeloproliferative disorders. Leukemia.

[27] Rapado I, Albizua E, Ayala R, Hernández JA, Garcia-Alonso L, Grande S, Gallardo M, Gilsanz F, Martinez-Lopez J (2008). Validity test study of JAK2 V617F and allele burden quantification in the diagnosis of myeloproliferative diseases. Ann Hematol.

[28] Lippert E, Girodon F, Hammond E, Jelinek J, Reading NS, Fehse B, Hanlon K, Hermans M, Richard C, Swierczek S, Ugo V, Carillo S, Harrivel V (2009). Concordance of assays designed for the quantification of JAK2V617F: a multicenter study. Haematologica.

[29] Cankovic M, Whiteley L, Hawley RC, Zarbo RJ, Chitale D (2009). Clinical performance of JAK2 V617F mutation detection assays in a molecular diagnostics laboratory: evaluation of screening and quantitation methods. Am J Clin Pathol.

[30] Chen Q, Lu P, Jones AV, Cross NC, Silver RT, Wang YL (2007). Amplification refractory mutation system, a highly sensitive and simple polymerase chain reaction assay, for the detection of JAK2 V617F mutation in chronic myeloproliferative disorders. J Mol Diagn.

[31] Denys B, El Housni H, Nollet F, Verhasselt B, Philippé J (2010). A real-time polymerase chain reaction assay for rapid, sensitive, and specific quantification of the JAK2V617F mutation using a locked nucleic acid-modified oligonucleotide. J Mol Diagn.

[32] Mason J, Akiki S, Griffiths MJ (2011). Pitfalls in molecular diagnosis in haemato-oncology. J Clin Pathol.

[33] Martinaud C, Brisou P, Mozziconacci MJ (2010). Is the JAK2(V617F) mutation detectable in healthy volunteers?. Am J Hematol.

[34] Sidon P, El Housni H, Dessars B, Heimann P (2006). The JAK2V617F mutation is detectable at very low level in peripheral blood of healthy donors. Leukemia.

[35] Xu X, Zhang Q, Luo J, Xing S, Li Q, Krantz SB, Fu X, Zhao ZJ (2007). Prevalence in a large Chinese hospital population. Blood.

[36] Nielsen C, Birgens HS, Nordestgaard BG, Bojesen SE (2013). Diagnostic value of JAK2 V617F somatic mutation for myeloproliferative cancer in 49 488 individuals from the general population. Br J Haematol.

[37] Passamonti F, Rumi E, Pietra D, Lazzarino M, Cazzola M (2007). JAK2 (V617F) mutation in healthy individuals. Br J Haematol.

[38] Lippert E, Mansier O, Migeon M, Denys B, Nilsson A, Rosmond C, Lodé L, Ugo V, Lascaux A, Bellosillo B, Martinez-Lopez J, Naguib D, Gachard N (2014). Clinical and biological characterization of patients with low (0.1–2%) JAK2V617F allele burden at diagnosis. Haematologica.

[39] Jovanovic JV, Ivey A, Vannucchi AM, Lippert E, Oppliger Leibundgut E, Cassinat B, Pallisgaard N, Maroc N, Hermouet S, Nickless G, Guglielmelli P, van der Reijden BA, Jansen JH (2013). Establishing optimal quantitative-polymerase chain reaction assays for routine diagnosis and tracking of minimal residual disease in JAK2-V617F-associated myeloproliferative neoplasms: a joint European LeukemiaNet/MPN&MPNr-EuroNet (COST action BM0902) study. Leukemia.

[40] WHO/BS/2016.2293 Collaborative study to evaluate the proposed WHO 1st International Reference Panel for Genomic JAK2 V617F :http://www.who.int/biologicals/ECBS_2016_BS2293_JAK2_WHO_reference_panel.pdf?ua=1

[41] Minervini A, Francesco Minervini C, Anelli L, Zagaria A, Casieri P, Coccaro N, Cumbo C, Tota G, Impera L, Orsini P, Brunetti C, Giordano A, Specchia G (2016). Droplet digital PCR analysis of NOTCH1 gene mutations in chronic lymphocytic leukemia. Oncotarget.

[42] Fontanelli G, Baratè C, Ciabatti E, Guerrini F, Grassi S, Del Re M, Morganti R, Petrini I, Arici R, Barsotti S, Metelli MR, Danesi R, Galimberti S (2015). Real-Time PCR and Droplet Digital PCR: two techniques for detection of the JAK2(V617F) mutation in Philadelphia-negative chronic myeloproliferative neoplasms. Int J Lab Hematol.

